# Making the climate crisis personal through a focus on human health

**DOI:** 10.1007/s10584-021-03107-y

**Published:** 2021-06-17

**Authors:** Vijay S. Limaye

**Affiliations:** Natural Resources Defense Council, New York City, NY USA

**Keywords:** Public health, Resilience, Epidemiology, Economic valuation, Mapping

## Abstract

Climate change–driven health impacts are serious, widespread, and costly. Importantly, such damages are largely absent from policy debates around the costs of delay and inaction on this crisis. While climate change is a global problem, its impacts are localized and personal, and there is growing demand for specific information on how climate change affects human health in different places. Existing research indicates that climate-fueled health problems are growing, and that investments in reducing carbon pollution and improving community resilience could help to avoid tens to hundreds of billions of dollars in climate-sensitive health impacts across the USA each year, including those stemming from extreme heat, air pollution, hurricanes, and wildfires. Science that explores the underappreciated local health impacts and health-related costs of climate change can enhance advocacy by demonstrating the need to both address the root causes of climate change and enhance climate resilience in vulnerable communities. The climate crisis has historically been predominantly conceived as a global environmental challenge; examination of climate impacts on public health enables researchers to localize this urgent problem for members of the public and policymakers. In turn, approaches to climate science that focus on health can make dangerous climate impacts and the need for cost-effective solutions more salient and tangible.

## Introduction

The climate crisis is often presented as an Earth system problem because increasing concentrations of greenhouse gasses are contributing to rising sea levels, increasing global temperatures, and accentuated extremes of the planet's hydrologic cycle (both extreme drought and extreme rainfall). While climate change is indeed a global crisis, its impacts are localized and personal. As this problem worsens, there is growing demand from members of the public and from policymakers for more specific information on climate change impacts to human health in different regions. Existing research indicates that climate-fueled health impacts are growing in severity and reach, and that investments in reducing carbon pollution and improving community resilience could help to avoid thousands of premature deaths and hospitalizations and tens to hundreds of billions of dollars in climate-sensitive health impacts across the USA each year, including those stemming from extreme heat, drought, air pollution, hurricanes, severe storms, and wildfires (Jay et al. 2018; Limaye et al. 2019). Science that explores the underappreciated local physical and mental health risks and health-related costs of climate change (stemming from climate-related injuries, illnesses, and deaths) can enhance advocacy efforts and policy solutions by demonstrating the need to both address the root causes of climate change and enhance climate resilience in vulnerable communities (Limaye et al. 2020b).

For more than two decades, public health researchers have been connecting the climate crisis to tangible human health risks through both direct and indirect mechanisms (Patz et al. 2000, 2014). Directly, climate change heightens human health risks from exposure to extreme heat, prolonged drought (a phenomenon linked to air pollution and mental distress), and extreme storms (associated with flooding and injuries) (Achakulwisut et al. 2018; Chakraborty et al. 2019), amongst other hazards. Indirectly, warmer ocean temperatures fuel stronger coastal storms (Marsooli et al. 2019), while warmer ambient air temperatures promote the formation of ground-level ozone smog air pollution and allow insect vectors to carry diseases like West Nile Virus and Lyme Disease to new geographies (Crimmins et al. 2016).

Climate change clearly threatens human health through both direct and indirect mechanisms, but still the health ramifications of the climate crisis remain understudied and underacknowledged. For example, a recent review found that health-focused research represented only about 1% of all climate change research in 2017 (Watts et al. 2018). Funding for interdisciplinary research at the intersection of climate change and human health is similarly anemic (Ebi et al. 2016). In public discussions, the lack of emphasis on climate change as a health problem persists, despite research indicating that a health frame motivates high levels of support for climate change mitigation and adaptation policies (Maibach et al. 2010; Nabi et al. 2018; Walker et al. 2018; Kotcher et al. 2018). In a recent study in North America, few members of the general public were able to identify *any* specific health problems linked to climate change, nor could they identify any at-risk populations, though respondents appeared to view the climate crisis as a threat to health (Hathaway and Maibach 2018). Health framing has been demonstrated as a proven motivator for undertaking personal actions on the climate problem (Amelung et al. 2019), and health concerns are important in shaping household decision-making related to climate impacts (Herrmann et al. 2020). Polling of USA public opinion in recent years demonstrates that the American public increasingly views climate change as an urgent threat that poses risks to well-being (Kotcher et al. 2020), though the COVID-19 pandemic has recently diminished support, at least temporarily, for climate action (Johnson 2020).

Despite remaining gaps in the science linking climate change to hazardous environmental exposures to health problems in specific populations, policymakers are grappling with the challenge of responding to the climate crisis because climate-fueled disasters with serious health impacts are increasingly visible and obviously damaging. Climate attribution science now allows scientists to demonstrate quantitative links between greenhouse gas concentrations, climate warming, and the observed uptick in severity of extreme events (van der Wiel et al. 2017; Imada et al. 2019) Quantitative determinations about the role of climate change in worsening weather can bolster public demands to address the underlying problem (Boudet et al. 2020).

Efforts to frame climate science in human health terms can improve the usability of this research for advocacy directed at policymakers because it can be presented in tangible, identifiable, and relatable terms (Spence et al. 2012; McDonald et al. 2015). Vague information on risks to to economic stability and property values are less convincing than threats to human health, especially those presented in psychologically proximate ways that link audiences to victims with low social distance (Hart and Nisbet 2014). But there remains the challenge of making climate change and health science more usable. Various health framing strategies can be employed by both scientific experts and advocates to improve the usability of climate science. Specifically, health frames that focus on human (rather than Earth system scales) in space and time, cumulative experiences of risks (rather than hazards considered in isolation from one another), and health risks and benefits presented in economic terms can achieve broader and more aggressive actions on mitigation and adaptation. This essay explores these strategies in detail and describes examples of their applications, with consideration made to both the benefits and limitations of health framing for improving the usability of climate science. Ultimately, a climate science approach focused on human health can help to make this vital information more relevant and interventions to improve resilience more urgent.

## Approaches

A health-centered approach to climate science helps to make the problem more salient to members of the public and policymakers. But even as public health research on climate-fueled risks accumulates, it is increasingly urgent for experts in climate change and health to synthesize, translate, and disseminate that information to a broad set of public stakeholders by deploying a variety of tools and engaging multiple communication channels (Keller and Limaye 2020).

Here, I describe three approaches that can improve the usability of climate science: (1) representation of climate impacts through health problems on human scales (both spatial and temporal), (2) representation of health problems through a cumulative impacts lens, and (3) translation of health problems related to climate change (and the health benefits of climate action) in economic terms. Collectively, these strategies have the potential to make the consequences of the climate crisis more tangible. Application of these approaches can also motivate stronger action to reduce climate-changing pollution and improve resilience through more aggressive adaptation efforts in the face of this unprecedented societal challenge.

### A human scale

Fundamentally, undertaking a human health–centered approach to communicating about the implications of the climate crisis is about bringing this issue to a more relatable scale. While international and national assessments of climate change, such as those produced by the United Nations Intergovernmental Panel on Climate Change (IPCC) and the US Global Change Research Program, are focused on describing broad climate mechanisms, risks, and trends, this information is of limited value to people seeking to understand what climate change could mean for them and their local communities specifically. Just as both local and global actions are needed to appropriately contend with the climate crisis, so too must information about the impacts of climate change be made available on multiple scales (Patz et al. 2005; Spangler et al. 2019). But an advantage of presenting climate change as a local and regional health problem is that such an approach lends itself to the field of applied epidemiology, which relates changes in local and/or regional environmental conditions (e.g., changes in daily minimum and maximum temperatures) to expected changes in the incidence of health problems (e.g., heat stroke) (Limaye et al. 2018).

Mapping tools are particularly useful for making the health problems of the climate crisis locally visible. Maps can be used as effective communication tools (Lahr and Kooistra 2010) because these tools allow people to visualize these within an accessible spatial context. In terms of climate-fueled health risks, these maps can also help to demonstrate the far-reaching nature of these challenges. For example, maps depicting the spread of wildfire smoke that travels far downwind from burned areas help to explain why even people who do not live in fire-prone areas are vulnerable to lung and heart disease stemming from air pollution in wildfire-generated smoke (Fann et al. 2018).

In contrast to static maps published in peer-reviewed journals and comprehensive climate assessment reports, web-based mapping platforms can facilitate dynamic and interactive mapping tools that allow users to explore localized impacts of climate change. For example, the Natural Resources Defense Council (NRDC) has published interactive climate change and health maps that demonstrate how extreme heat and air pollution exposures are distributed around the USA (Natural Resources Defense Council 2017). These maps make use of publicly available data through a platform that allows users to "zoom in" from a broad view to understand local trends at the county level, and the health implications of these climate-sensitive exposures. Importantly,  simply enabling people to access such mapping tools does not always engender stronger support of climate change response policies. For example, some researchers have detected reduced levels of climate concern among users of sea level rise maps for people who themselves already acknowledge climate risks (Mildenberger et al. 2019). Expanded evaluation of how maps can best inform stakeholders, taking into account efficacy of knowledge transfer and impacts on end-user understanding (Wong-Parodi et al. 2014), is needed to further refine these decision support tools.

Human storytelling around climate change and health can also expose audiences to compeling information sourced directly from people impacted by climate hazards. For example, the Climate Literacy and Energy Awareness Network has inventoried and reviewed videos that demonstrate these links and serve as another medium to communicate research findings (Climate Literacy and Energy Awareness Network 2020). Similarly, NRDC has developed multiple videos on climate and health topics that interweave first-person accounts with peer-reviewed scientific information in a complementary way (Limaye 2019, 2020).

### Trends and synthesis

A second way to make the available climate and health science more usable is to compile and synthesize it with regular frequency. While IPCC climate reports present global summaries, these reports are published relatively infrequently in part due to their global reach, complexity, and robust authorship and peer review processes. As a result of these infrequent updates and a focus on identifying climate trends rather than changes in projected health risks, as climate harms to health continue to mount, the IPCC role in communicating the links between climate and health unfortunately remains limited. A similar problem afflicts the US National Climate Assessment, which is mandated for production on a 5-year cycle (Crimmins et al. 2016; U.S. Global Change Research Program 2018). Efforts have been made in recent years to more rapidly synthesize the available climate and health science, including through the Lancet Countdown on Climate Change and Health and accompanying country-specific briefs (Salas et al. 2019; Watts et al. 2019, 2020). The combination of global- and national-level information presented in these assessments can be supplemented with regional- and state-level synthesis issue briefs (Constible 2019) that facilitate expanded examination of these impacts within distinct geographies.

In addition to more frequently summarizing the available climate and health evidence, it is also helpful to consider recent data within the historical context and report that information at a local scale. For example, current web tools allow users to input a zip code and understand temperature trends over recent decades during one’s own lifetime (Climate Central 2019). Such an approach allows people to understand the process of climate change on human time scales (years to decades), rather than the extended time horizons typically deployed in large climate assessment reports, which typically describe environmental changes at 10-, 50- or 100-year scales. Historical context is particularly important because research shows that people may adjust to a “new normal” of local weather conditions and lose sight of significant long-term trends (Moore et al. 2019).

The benefits of synthesizing climate information can also intersect with the application of analysis at a human scale. Cumulative impacts framing can allow people to understand how discrete exposures are typically experienced (e.g., the combined burden of extreme heat and summertime ozone air pollution in urban areas). A cumulative impacts approach can help to demonstrate the inequitable burden of harmful exposures (Mikati et al. 2018; Colmer et al. 2020) that may be legally permitted because of regulatory frameworks that do not consider the synergistic effects of multiple, combined pollution exposures. This approach can also help to quantify the complex effects of climate change on health (e.g., the unintended air pollution and public health consequences of cooling adaptations such as air conditioning units powered at least in part by electricity generated from fossil fuel combustion) (Abel et al. 2018). As epidemiologic evidence on the synergistic effects of exposures grows (Ren et al. 2008; Krug et al. 2019), it is important that climate and health communications reflect the human experience of this problem and the importance of compounding vulnerability and exposure factors that confer disproportionate health burdens on marginalized groups.

### Economic ramifications of climate-sensitive problems

Climate change–driven health impacts are serious, widespread, and costly but such damages are largely absent from policy debates around the costs of delay and inaction on this crisis. Economic valuation of climate-sensitive health problems (Knowlton et al. 2011; Limaye et al. 2019; Patz et al. 2020) translates health problems into economic terms by estimating the personal and societal toll of premature deaths, hospitalizations, emergency room visits, and ongoing medical care needs stemming from climate risks. Given the high degree of medical financial hardship in the USA (Yabroff et al. 2019), an economic valuation approach can explain how the climate crisis intersects with systemic racism and an inequitable health insurance system. There is also evidence that presenting individuals with information about the projected adaptation costs associated with unchecked climate change can result in a small increase in willingness to support climate change mitigation policies (Carrico et al. 2015; Greenhill et al. 2018). Science that describes adaptation interventions that improve health can be communicated in economic terms (Hess et al. 2018) to better make the case for cost-beneficial investments in community preparedness and other efforts to bolster resilience to climate harms.

The economic framing of climate change damages to health can also be reoriented to describe the health and financial benefits of climate action (Limaye et al. 2020b). This approach makes clear the financial benefits of climate response policies that reduce adverse (and sometimes irreversible) health impacts and costly medical care in hospitals and emergency rooms. Such information can be utilized by governments at the federal, state, and local levels to justify the investment of resources and staffing to execute climate adaptation projects aimed at public health protection (Hess et al. 2018). Published evidence indicates that climate change mitigation policies can achieve near-term health improvements across multiple sectors, including active transportation (Grabow et al. 2012), household energy (Wilkinson et al. 2009), and diet (Springmann et al. 2016; Stull and Patz 2020). In a similar fashion, benefits analyses tied to climate goals articulated in specific international agreements (Lo et al. 2019) may help to articulate the local benefits of concerted global action and strengthen solidarity to support sustained mitigation actions over time.

## Benefits and limitations

Collectively, the three strategies described here (use of human scales, a cumulative impacts lens, and economic framing) have the potential to improve the salience of climate science for both members of the public and government leaders. These approaches enhance the advocacy case for climate action and the evidence base for climate policies that protect health, particularly for building support for climate action by engaging health professionals as advocates (McCarthy and Bernstein 2019; Maibach et al. 2019). Moreover, stakeholders can make use of the substantial scientific evidence on climate-sensitive health effects to inform health protective policy solutions and slow the accumulation of health harms, without relying on statistical attribution of climate change to specific health harms, which is still years away (Ebi et al. 2017).

Nevertheless, there are key limitations and obstacles to consider in applying these strategies. First of all, public health practitioners have to date been poorly integrated into climate policy planning efforts (Sheehan et al. 2017; Fox et al. 2019), even though public health data systems and staff expertise in deploying community interventions can materially advance such work. Second, achieving effective communication of climate and health information remains a challenge. Communication problems and the deployment of scientific jargon (Shulman et al. 2020) seriously afflict climate science *and* public health science, and prevent members of the public from accessing key information about the major impacts of climate change on health (Albright et al. 2020). Third, limited public health funding for climate work (Himmelstein and Woolhandler 2016) inhibits production of useful information at the state and local level. Fourth, it is not clear that crucial information on the clear links between climate change and health is adequately represented in educational curricula, though recent efforts have been made to consolidate a comprehensive educational approach to this issue at the professional training level (Shaman and Knowlton 2018). More consistent and robust teaching of this topic through a climate and health literacy framework (Limaye et al. 2020a) can help to enhance uptake of this important information for students in K-12 and undergraduate settings. Fifth, in terms of identifying inequitable climate impacts on health, gaps in available data limit the potential of research to support and supplement climate-health storytelling with empirical evidence. For example, identification of inequitable cost burdens of climate-related health problems shouldered by marginalized groups, including communities of color and low-income communities, is currently hampered by poor data availability (Limaye et al. 2019). Similarly, the ethical ramifications of inequitable climate-related harms are difficult to characterize at local level (Patz et al. 2007; Pearson et al. 2018) and data limitations similarly preclude adequate identification of gender differences in climate impacts on health (Rao et al. 2019). While our understanding of climate links to other problems like mental health (Lõhmus 2018; Obradovich et al. 2018), drought (Achakulwisut et al. 2018), and displacement (Cash et al. 2020) is limited, we still need to account for these harms in real time in order to avoid worsening damages to human health.

## Conclusion

The climate crisis has historically been predominantly conceived as a global environmental challenge. Examination of growing climate harms to public health, considered within the global public dialogue and debates amongst policymakers, can enable people to recognize major personal risks tied to this urgent societal crisis. In turn, approaches to climate science that focus on implications for health can make dangerous and expensive climate risks (and cost-effective mitigation and adaptation solutions) more salient and tangible to members of the public and government leaders.
